# Screening of Lactic Acid Bacteria for the Bio-Control of *Botrytis cinerea* and the Potential of *Lactiplantibacillus plantarum* for Eco-Friendly Preservation of Fresh-Cut Kiwifruit

**DOI:** 10.3390/microorganisms9040773

**Published:** 2021-04-07

**Authors:** Nicola De Simone, Vittorio Capozzi, Maria Lucia Valeria de Chiara, Maria Luisa Amodio, Samira Brahimi, Giancarlo Colelli, Djamel Drider, Giuseppe Spano, Pasquale Russo

**Affiliations:** 1 Department of Agriculture, Food, Natural Science, Engineering, University of Foggia, Via Napoli 25, 71122 Foggia, Italy; nicola.desimone@unifg.it (N.D.S.); m.dechiara@unifg.it (M.L.V.d.C.); marialuisa.amodio@unifg.it (M.L.A.); giancarlo.colelli@unifg.it (G.C.); giuseppe.spano@unifg.it (G.S.); 2 Institute of Sciences of Food Production, National Research Council of Italy (CNR), c/o CS-DAT, Via Michele Protano, 71121 Foggia, Italy; vittorio.capozzi@ispa.cnr.it; 3 Laboratory of Applied Microbiology, Department of Biology, Faculty of Natural Sciences and Life, University of Oran 1 Ahmed Ben Bella, Bp1524 El M’ Naouer, Oran 31000, Algeria; sam90.brahimi@gmail.com; 4 UMR Transfrontalière BioEcoAgro1158, Univ. Lille, INRAE, Univ. Liège, UPJV, YNCREA, Univ. Artois, Univ. Littoral Côte D’Opale, ICV-Institut Charles Viollette, 59000 Lille, France; djamel.drider@univ-lille.fr

**Keywords:** *Botrytis cinerea*, post-harvest, kiwifruit, lactic acid bacteria, antifungal activity, bio-control, sustainability

## Abstract

*Botrytis cinerea*, responsible for grey mold, represents the first biological cause of fruit and vegetable spoilage phenomena in post-harvest. Kiwifruit is a climacteric fruit particularly prone to this mold infestation during storage. Lactic acid bacteria (LAB) are food-grade bacteria that can synthesize several metabolites with antimicrobial activity and are, therefore, suggested as promising and eco-friendly resources for the bio-control of molds on fruits and vegetables. In this work, we propose the screening of a collection of 300 LAB previously isolated from traditional sourdoughs for their ability to counteract in vitro the growth of *Botrytis cinerea* CECT 20973. Only 2% of tested LAB strains belonging to *Lactiplantibacillus plantarum* species, exerted a strong antagonism against *B. cinerea*. The cell-free supernatants were partially characterized and results clearly indicated that high levels of lactic acid contributed to the antagonistic activity. PAN01 and UFG 121 cell-free supernatants were investigated as potential bio-control agents in a preliminary in vivo assay using freshly cut kiwifruits as a food model. The application of cell-free supernatants allowed to delay the growth of *B. cinerea* on artificially contaminated kiwifruits until two weeks. The antagonistic activity was greatly affected by the storage temperature (25 °C and 4 °C) selected for the processed fruits, suggesting the importance to include microbial-based solution in a broader framework of hurdle technologies.

## 1. Introduction

Consumers highly appreciate kiwifruit for its sour and sweet taste, but also for its associated healthy features, attributed to high levels of vitamin C, flavonoids, minerals, and significant amounts of chlorophyll and carotenoids. Its consumption, as part of a balanced diet, has been reported to have multiple benefits (other than the nutritional ones), such as antioxidant capacity [1], an increase of HDL cholesterol and decrease of triglycerides [2], protection from oxidative stress [3], and favorable changes in the human colonic microbial community [4]. In addition, it is a crop with high economic relevance, with about four million tons produced worldwide in 2017, and approximately 1.5 million tons exported among countries [5]. The most important world kiwifruit producers are China, Italy, New Zealand, Chile, and Greece, representing more than 85% of the global production [1].

Kiwifruit is a climacteric fruit that can be stored for several months if harvested at physiological maturity. Recommended conditions include storage temperature of 0 °C, relative humidity higher than 90% and atmosphere modification with 1 to 2% O_2_ and 3 to 5% CO_2_. Removal of ethylene from the storage atmosphere is also crucial [6]. Limiting factors for long-term storage is the loss of firmness due to ripening, especially in presence of ethylene, even at very low concentrations, and development of fungal decay. Removal of ethylene from storage rooms using ethylene scrubbers is highly recommended, but also treatment with 1-methylcyclopropene, a powerful inhibitor of its action, is very effective in slowing down fruit softening [7].

Among spoilage microorganisms, filamentous fungi are well-known to affect a wide range of fruits commodities, leading to significant economic losses and food wastes. Furthermore, they are considered as allergens, and they could produce mycotoxins, posing a critical concern for the safety of the consumers [8]. *Botrytis cinerea*, the grey mold’s etiological agent, is a typical contaminant of a wide range of fruits and vegetables, with high relevance in terms of economic impact [9]. In particular, *B. cinerea* is the primary filamentous fungus responsible for the decay of kiwifruits [10], causing dark green color, water-soaked and soft texture on diseased tissues [11]. Although several methods are reported for the bio-control of grey mold in other commodities [12], only a few data are available for kiwifruits. Indeed, this disease is mainly controlled in the field and in the cold room by using synthetic fungicides. However, the appearance of fungicides-resistant strains [13], and the growing consumers demand for residue-free products has encouraged producers to adopt integrated management of post-harvest grey mold on fruit crops [14].

In recent years, with the aim to provide eco-friendly and safer approaches for the control of undesired microflora in food commodities, the use of microbial-based solutions is increasing [15]. Among these, the employment of Lactic Acid Bacteria (LAB) capable of inhibiting spoilage microorganisms can be a preferable choice because of the long history of safe use testified by the status of “generally recognized as safe” (GRAS) and “qualified presumption of safety” (QPS) [16]. Since LAB can be deliberately added into the food chains, in the last years, several studies suggested the use of LAB with antimicrobial properties as natural food preservatives against the development of spoilage fungi [17,18]. Their activity is expressed both through the competition for fundamental nutrients, and as a result of the synthesis of active compounds, such as organic acids, carbon dioxide, ethanol, hydrogen peroxide, fatty acids, acetoin, diacetyl, cyclic dipeptides, bacteriocins, or bacteriocin-like inhibitory substances [17,19]. Nevertheless, synergistic mechanisms among different metabolites have been reported to increase the overall antimicrobial activity substantially [20]. In addition, the availability of new analytical approaches is facilitating the purification and the characterization of new preservative compounds [21]. For these reasons, the discovery of new antifungal molecules and the understanding of the molecular basis of microbial interactions are receiving growing effort by the researchers.

In the fresh-cut sector, epiphytic bacteria and/or food-grade LAB strains have been proposed for the biocontrol of foodborne pathogenic bacteria (i.e., *Salmonella* spp., *Escherichia coli* O157:H7, and *Listeria monocytogenes*) [22,23], while molds are mainly controlled with physico-chemical approaches [24], or the addition of bioactive polyphenols [25,26]. In a recent work, two strains of *Lactiplantibacillus plantarum* have been incorporated into a Konjac-based edible coating in order to prevent the growth of naturally occurring mold and yeast [27], suggesting the potential of this microbial species as biocontrol agent.

However, to the best of our knowledge, no studies report on the use of microbial metabolites produced from LAB for the bio-control of kiwifruits. For this reason, this work aims (i) to select LAB strains capable of inhibiting *Botrytis cinerea*; (ii) to partially characterize the active compounds responsible for the antifungal activity; and (iii) to assess the in vivo activity by using freshly cut kiwifruits as fruit model.

## 2. Materials and Methods

### 2.1. Microbial Strains and Growth Conditions

Three-hundred LAB strains previously isolated from five different artisanal sourdoughs [18,28], and available at the culture collection of the Industrial Microbiology Laboratory of the University of Foggia, were routinely cultured in MRS broth (Oxoid, Basingstoke, UK) at 30 °C.

*Botrytis cinerea* CECT 20973 was obtained by the Spanish Type Culture Collection (CECT, Paterna, Spain), and utilized as target spoilage strain in the screening assay. Lyophilized culture was re-suspended on saline solution (0.9% NaCl), plated on Potato Dextrose Agar (PDA, Oxoid), and incubated at 25 °C for 5 days. Fungal spore suspension was prepared by brushing the plate surface with saline solution using a sterile swab and stored at 4 °C up to a maximum of 7 days. During this time, the fungal spores concentration was steady, as determined by plating serial dilution on PDA plates.

### 2.2. Screening of Antifungal Activity

The overlayed method was used for a fast initial screening of the antifungal activity of LAB strains against *B. cinerea* [28]. Briefly, 5 µL of cultures at late exponential phase (approximately 16-h of incubation, according to previously generated standard growth curves) were spotted on MRS agar plates and incubated at 30 °C for 24 h. Then, plates were overlaid with 10 mL of Malt Extract (Oxoid) Soft Agar (0.75% agar) inoculated (1:100 *v*/*v*) with a suspension containing approximately 1 × 10^6^ spores/mL of *B. cinerea*. After 3 days of incubation at 25 °C, LAB strains were discriminated on the basis of the halo of inhibition surrounding the spots and classified as strains of no (−), mild (+), or strong (++) inhibition, showing inhibition zone lower than 1 mm, ranging from 1 to 5 mm, or more than 5 mm, respectively. Assays were performed in duplicate.

### 2.3. Molecular Identification of LAB Species

The LAB strains showing the best antifungal performance (inhibition halo higher than 10 mm) were identified by sequencing of 16S rRNA. Genomic DNA was extracted by using the UltraClean^®^ Microbial DNA Isolation Kit, according to the manufacturer’s instruction (Qiagen, Hilden, Germany), and the 16S rRNA gene amplified by using BSF8 (5′-AGAGTTTGATCCTGGCTCAG-3′) and BSR1541 (5′-AAGGAGGTGATCCAGCCGCA-3′) primers. PCR was performed in 20 µL reaction volume, containing 2 µL buffer 10X, 4 µL buffer Q, 0.4 µL dNTP mix (10 µM), 0.5 µL for each primer, 0.1 µL Taq polymerase (Qiagen). The PCR protocol was as follows: denaturation at 94 °C for 4 min, followed by 34 cycles of denaturation at 94 °C for 30 sec, annealing at 56 °C for 30 sec, elongation at 72 °C for 90 sec, and a final extension at 72 °C for 5 min. Amplicons were analyzed by electrophoresis (90 V, 30 min) on 1% agarose gel containing ethidium bromide, visualized by UV fluorescence, and sequenced (Macrogen, Madrid, Spain). Results were submitted for comparison with sequences available at the NCBI database (GenBank) using the standard nucleotide–nucleotide homology search Basic Local Alignment Search Tool.

### 2.4. Partial Characterization of the Antifungal Activity of LAB Cell-Free Supernatant

The LAB strains showing strong antifungal ability were grown until the late exponential and stationary phase by incubation in MRS at 30 °C for 24 and 48 h, respectively. Growth curves were determined by monitoring the optical density at 600 nm (OD_600_) for 48 h using the plate reader BioTek Eon spectrophotometer (BioTek, Winooski, VT, USA). The corresponding cell-free supernatants (identified as CFS24 and CFS48) were obtained by centrifugation (8,000× *g* × 5 min) and filtration (0.45 µm-pore-size filter; VWR international, West Chester, PA, USA). In order to partially characterize the metabolites responsible for the antagonistic activity, an aliquot of the CFSs was neutralized (pH = 7) by adding KOH 1 M (Sigma-Aldrich, St. Louis, MO, USA). The antifungal activity of CFSs was determined by using the method of radial growth inhibition of hyphae [29]. Briefly, PDA plates were supplemented with 20% (*v*/*v*) of CFSs, and 10 µL of a freshly-prepared solution containing approximately 1 × 10^6^ spores/mL of *B. cinerea* were spotted at the center of the plate. The control plates were prepared by adding the same concentration of sterile MRS broth. Inhibition percentage was determined by measuring the radial growth of the hyphae after 24, 48, and 72 h of incubation at 24 °C. All the assays were performed in triplicate.

### 2.5. Quantification of Lactic Acid

LAB strains were inoculated from cryopreserved stock (1:1000 *v*/*v*) in MRS, and aliquots of cultures were collected at 6, 24, 30, and 48 h of growth, centrifuged and filtered as above. Then, the pH was measured (Mettler Toledo, Columbus, OH, USA), and the amount of both L-lactic acid and D-lactic acid determined spectrophotometrically in a plate reader (BioTek) by using a specific enzymatic kit (Biogamma, Rome, Italy) according to manufacturer’s instructions. Three different biological and five technical replicates were carried out.

### 2.6. Fruit Decay Assay

Healthy “Hayward” kiwifruits (*Actinidia chinensis* var. *deliciosa* A. Chevalier) were purchased in a local market, washed twice with sterile distilled water and dried. After drying, fruits were cut and divided into similar pieces by using a sterile lancet. For the assay, kiwifruit pieces were artificially contaminated or not with *B. cinerea* at a concentration of about 1 × 10^6^ spores/mL (freshly-prepared) by dipping for 30 s in MRS (control), MRS containing Fenhexamid 4 mg/L (Teldor Plus, Bayer, Leverkusen, Germany), or in the CFS48 from PAN01 and UFG 121. Then, fruits were air-dried under a laminar flow hood. After drying, three pieces of kiwifruits for each treatment were stored in Petri dishes at 25 °C for 3 days or at 4 °C for 14 days. Each treatment was performed in triplicate. The decay development was monitored daily through image acquisition by using a vision computer system equipped with a digital color camera (EOS 00D, Canon, Melville, NY, USA) located vertically on a matte black background at a distance of 0.45 m [28].

### 2.7. Sensorial Quality Analysis

A group of ten trained panelists performed the sensory evaluations of kiwifruits at the end of the storage. Before evaluations, panelists were trained in order to recognize and score the quality attributes. Color, mold occurrence, overall acceptance, visual quality, and freshness were evaluated using a hedonic scale from 1 to 5, where 1 = not edible/100% mold presence, to 5 = very fresh/0% mold presence, with 3 fixed as limit of marketability.

### 2.8. Statistical Analysis

The distribution of data was analyzed by using the Kolmogorov–Smirnov test of normality. One-way ANOVA using StatGraphics Centurion XVI.I (StatPoint Technologies Inc., Warrenton, VA, USA), applying Tukey’s test was used to determine any statistically significant difference (*p* ≤ 0.05).

## 3. Results and Discussion

### 3.1. Screening of the Antifungal Activity of Lactic Acid Bacteria

LAB from sourdoughs have been widely investigated for their antifungal activity [30,31,32], and successfully proposed for increasing the shelf-life of bakery products [18,33,34,35,36]. In this work, we investigated the effectiveness of 300 LAB strains isolated from traditional sourdoughs to contrast *B. cinerea*, a specific and diffused spoilage microbe of fruits and vegetables.

The LAB strains mainly showed only a poor or modest ability to inhibit the fungal target, using the overlayed method. In fact, based on the inhibition halo, it was found that 98% of the tested LAB strains were barely able to inhibit *B. cinerea* CECT 20973 (inhibition halo lower than 3 mm), while only six strains (2%) exerted a strong antagonism, showing an inhibition halo higher than 10 mm. This result agrees with the analysis of the antifungal activity performed by Cheong et al. [37], which screened about 900 LAB strains, observing that only 12 isolates (less than 2%) had a strong antagonistic activity against common cheese spoilage molds belonging to the genera *Aspergillus*, *Penicillium,* and *Cladosporium*, suggesting that the ability to deeply counteract the development of specific fungal targets can be a trait not widely diffused among LAB (probably species- and strain-dependent), regardless from the food matrices.

The strains with the best antifungal performances were selected for further in vitro characterization and identified by sequencing the 16S rRNA (Table 1). The molecular analysis revealed that all the strains belonged to *Lactiplantibacillus plantarum* species (formerly *Lactobacillus plantarum*) [38]. This evidence confirmed the general antimicrobial potential of *L. plantarum* species [39,40,41] and the rising evidence of a possible contribution of lactobacilli, particularly *L. plantarum*, in the microbial-based bio-control activity against *B. cinerea* on fruits [27,42,43,44].

### 3.2. Anti-Botrytis Activity of Cell-Free Supernatants

**Figure 1:**
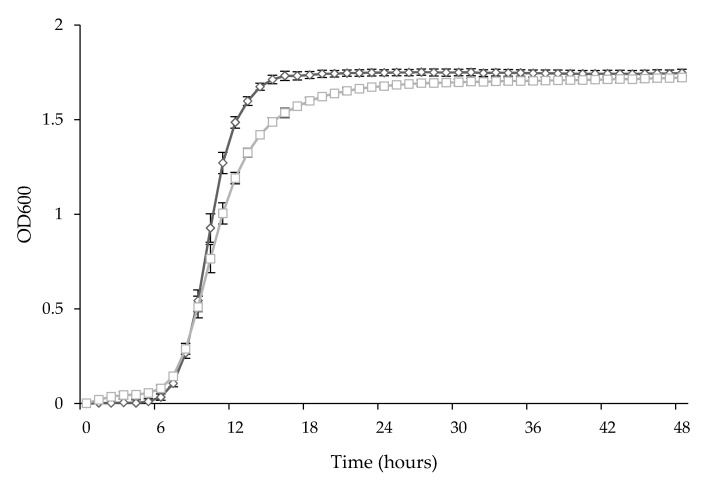
Growth curves of *L. plantarum* UFG 121 (square) and *L. plantarum* PAN01 (diamond). Data shown are mean ± SD (standard deviation) of three independent experiments.

In order to determine if the antifungal activity was due to direct antagonism or to the production of some metabolites, the CFSs were collected after 24 (CFS24) and 48 (CFS48) hours of incubation, a time corresponding to the late exponential and late stationary phase, respectively (an example is shown in Figure 1). The pH of each CFS was measured, as reported in Table 2.

**Figure 2:**
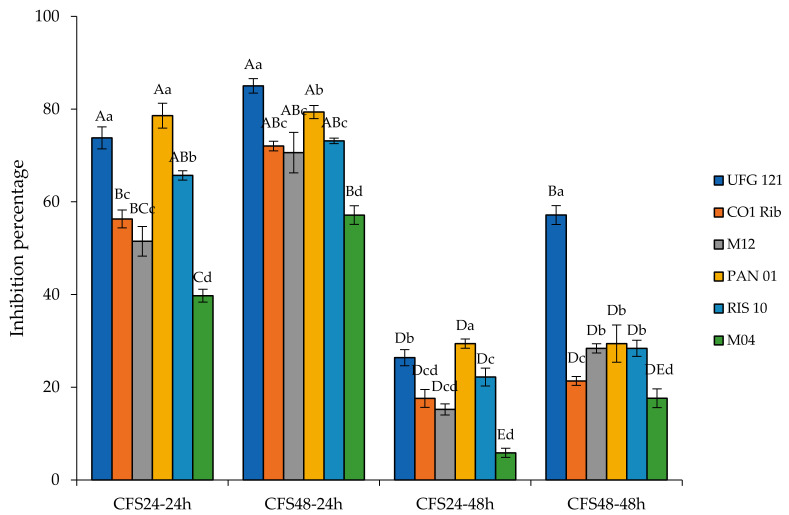
Hyphal radial growth inhibition of *B. cinerea* after 24 h and 48 h of incubation at 25 °C on plates of malt extract agar supplemented with 20% of CFS24 and CFS48 of the selected LAB strains. Capital letters indicate significant differences among the inhibition percentage of CFSs at all the experimental conditions. Lowercase letters indicate significant differences among the inhibition percentage of CFSs inside each experimental condition.

The inhibition was monitored based on the hyphae’s radial growth in plates containing or not 20% of the different CFSs, as described by Wang et al. [29]. As shown in Figure 2, the inhibition detected after 24 h of incubation dropped by half after 48 h, while it was not detectable after 72 h, indicating that the tested strains were able to delay at different extents, but not to inhibit completely, the growth of *B. cinerea* CECT 20973.

In detail, CFSs24 inhibited *B. cinerea* CECT 20973, between a minimum of 40% (M04) up to a maximum of about 80% (PAN01), after 24 h of mold growth. At this time of incubation, the antifungal activity of the CFSs48 of each *L. plantarum* strain notably increased, probably due to a further reduction of the corresponding pH. However, after two days of incubation, the ability to contrast the *B. cinerea* CECT 20973 growth was strongly reduced, ranging between 5 and 30% of inhibition for CFSs24 of M04 and PAN01, respectively. Interestingly, only CFS48 of *L. plantarum* UFG 121 showed a high inhibition capability after two days of the fungal growth (about 60%), which was almost double than what was observed for the CFSs48 of the other strains. These findings extended the knowledge concerning antimicrobial applications of CFSs from *L. plantarum* strains. In fact, several studies reported the potential of *L. plantarum* cell-free supernatants against foodborne pathogens (e.g., *Escherichia coli*, *Pseudomonas aeruginosa*, and *Staphylococcus aureus* [45,46,47]), other human pathogens (e.g., oral *Candida* species [48]), microbes producers of toxic compounds in foods (e.g., fumonisin producing *Fusarium proliferatum*, amine-positive bacteria [49,50]), spoilage fungi (e.g., *Aspergillus niger*, *Aspergillus flavus*, and *Fusarium oxysporum* [51,52]), and plant pathogens (e.g., *Pseudomonas syringae* pv. *actinidiae*, *Xanthomonas arboricola* pv. *pruni*, and *Xanthomonas fragariae* [53]). Additionally, few studies delved into the application of *L. plantarum* to limit the impact of *B. cinerea* as a fungal plant pathogen [29,54]. On the opposite, for the first time, the present study investigated the limitation of *B. cinerea* for post-harvest application on fruits/vegetables.

In general, our results showed some relationship between lower pH and higher antifungal activity, suggesting that an early production of organic acids might be responsible for the detected antagonism. Accordingly, in line with the evidence reported by Daranas et al. [53], when the assay was performed by using the corresponding neutralized CFSs, no inhibition was detected, corroborating the hypothesis that the antifungal activity is due to the production of organic acids. These compounds are the main products of the LAB’s secondary metabolism, and they are well-known for their preservative effectiveness [55,56]. However, it is noteworthy to point out that the pH of all CFSs48 were almost similar, except for M04 strain (that always showed the lowest inhibition), suggesting that a different composition of organic acids and/or the production of other compounds could be involved in the antifungal activity [57].

Thus, lactic acid production was monitored during 48 h in *L. plantarum* UFG 121 and *L. plantarum* PAN01, selected as the best antagonist strains (Table 3). Since *L. plantarum* can produce both L-lactate and D-lactate, each enantiomer was detected. After 24 h of growth, L-lactic acid production was slightly higher for strain UFG 121 than PAN01 (about 17 and 14 g/L, respectively). This difference further rose until achieving a final amount of about 27 and 20 g/L in 48 h-old cultures. Interestingly, L-lactate was the dominant form, corresponding to a concentration higher than 85% of the total. Moreover, the fraction of D-lactate produced by strain 121 was about double that of strain PAN01.

Similarly, the antagonistic strain *Lactiplantibacillus pentosus* LOCK 0979, a species very close to *L. plantarum*, produced 93% of L-lactic acid in MRS. However, in presence of sorbitol and galactosyl-polyols, the amount of D-lactate increased up to a concentration higher than 30% [58]. Interestingly, recent works report a rise of the antifungal activity in association with different polyols that was related to significant modification of the acid metabolite profile of the bacterial culture supernatant [59,60]. Although our results are in apparent contrast to what reported in Table 2, since the higher production of lactic acid observed for *L. plantarum* UFG 121 corresponded to a high pH of this strain than PAN01, the occurrence of other organic acids, probably less effective against *B. cinerea*, could determine the lower pH value observed for PAN01. On the other hand, it is well known that antagonistic compounds might contribute to the overall inhibition by having a synergistic or additive effect [61]. For example, as previously determined by Russo et al. [28], UFG 121 strain produced low amounts of phenyllactic acid, a compound displaying a broad spectrum of antifungal activity [20], which might partially explain this apparent incongruence. Indeed, it is presumed that the behavior of the antifungal activity is positively related to the phenyllactic acid and 4-hydroxyphenyllactic acid content in LAB culture filtrates [62]. However, a wide variety of other volatile organic acids (mainly including acetic, propanoic, butanoic, octanoic, and hydroxyl acids) have been suggested as active against *B. cinerea*, and the microorganisms responsible for their production proposed as biocontrol agents on strawberry, vine, and grape berries [63,64,65].

### 3.3. Anti-Botrytis Activity on Cut Kiwifruits

In order to investigate the potential of *L. plantarum* strains as protective treatment to delay the decay during storage of fruit commodities, CFS48 of *L. plantarum* PAN01 and UFG 121 strains were tested for a preliminary in vivo assay by using freshly cut kiwifruit as a food model. CFS were applied by dipping, a process usually employed to transfer antimicrobial, antibrowning, or texture preservative compounds to fresh-cut products. A fungicide (e.g., Fenhexamid) classified as a minimal risk to human health and environment for the control of grey mold in pre- and post-harvest was used to compare the efficacy of the proposed approach. Kiwifruits were stored for 3 days at 25 °C, to mimic a thermal abuse that could encourage the development of the spoiler, and at 4 °C for 14 days, simulating a correct management of the cold chain.

**Figure 3:**
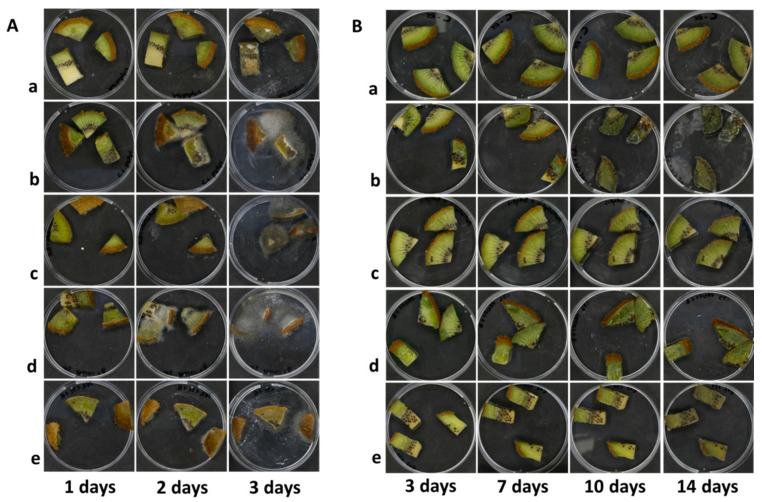
Image acquisition of kiwifruit pieces non- (**a**) or artificially contaminated with *B. cinerea* CECT 20973 (**b**) and treated with Fenhexamid (**c**), CFS48 of *L. plantarum* PAN01 (**d**), or UFG 121 (**e**), and stored for 3 days at 25 °C (**A**) or for two weeks at 4 °C (**B**).

As expected, when the assay was carried out at room temperature (Figure 3A) a fast development of *B. cinerea* was detected on all the artificially contaminated samples, although the fungal growth seems to be delayed in kiwifruits treated with CFS48 from UFG 121 strain and the chemical.

Under cold storage conditions (Figure 3B), *B. cinerea* began to develop only after the seventh day in non-treated contaminated samples, covering the whole fruit’s surface on the tenth day. In contrast, molds occurrence was only minimally detectable after two weeks in kiwifruits submitted to CFS48 treatments. No fungal growth was disclosed in control and chemically-treated samples.

**Figure 4:**
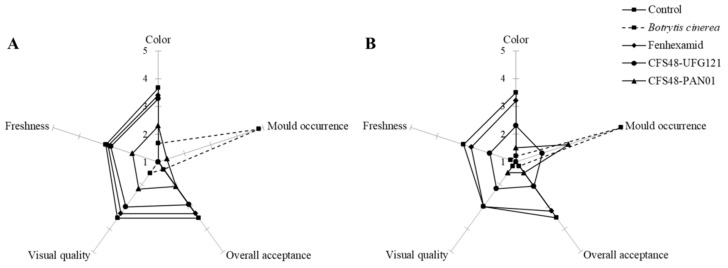
Sensorial evaluation of freshly cut kiwifruits after 10 (**A**) and 14 days (**B**) of storage at 4 °C.

In particular, kiwifruits stored at 4 °C were subjected to several modifications, such as color changes, loss of firmness, dehydration of the cut surfaces, probably associated with alterations in nutritional and organoleptic quality, started to occur after 10 days of preservation, regardless of contamination with *B. cinerea* (Figure 3B and Figure 4). These changes could be induced by biochemical reactions associated with cell senescence, accelerated by unit operations, such as cutting and washing.

Figure 4 show changes in sensory parameters after 10 days and at the end of storage time in cold-stored kiwifruits. As expected, it was observed that artificial contamination of kiwifruit pieces with *B. cinerea* greatly affected the product’s quality. Control and treated with Fenhexamid samples showed the best performance during storage, being still marketable after two weeks. At the same time, kiwifruits treated with CFS48 from *L. plantarum* UFG 121 showed better behaviors than PAN01 reaching not acceptable overall quality anyhow. Interestingly, after 10 days of cold storage, the kiwifruit pieces dipped in CFS48 of UFG 121 were considered to be of sufficient quality for marketing, as no significant differences were found with the control fruits.

This time is consistent with what observed for fresh-cut pear submitted to a postharvest calcium supplementation with *Lactobacillus rhamnosus* GG [66], while coated kiwi slices loaded with probiotic *L. plantarum* retained overall acceptability and physicochemical characteristics for five days of cold storage [29].

Therefore, our results suggest that, despite the LAB strains analyzed might not be used for applications in which a complete inhibition of *B. cinerea* is required, they could still be valuable in the design of protective microbial-based solutions to delay its growth, extending shelf life and improving fruit marketability. In particular, it was further confirmed the broad antifungal activity of strain UFG 121 in different food matrices [17,28], indicating potential applications also in the biocontrol of fruit products. Moreover, in this study, conditions encouraging fungal contamination, including a high level of spores and packaging in passive atmosphere, have been evaluated, suggesting a potential greater biocontrol effectiveness than what was observed. Accordingly, it has been reported that that the antimicrobial effectiveness of live bacteria on fresh-cut fruits was positively correlated with antagonist concentration [67,68]. However, the addition of viable bacteria could drive detrimental fermentations, leading to off-flavors’ production impacting the overall quality of the fruits [68].

## 4. Conclusions

Filamentous fungi are responsible for significant food deterioration, as well as safety concerns due to their potential ability to produce mycotoxins. In recent years, LAB have been extensively studied for their ability to counteract the fungal growth, and they can be considered as a promising strategy for the bio-conservation of fruits and vegetables [69]. In particular, increasing shelf life without the addition of chemical additives is one of the main challenges for the sector. In this work, we selected two *Lactiplantibacillus plantarum* strains from a large cohort of LAB based on their ability to contrast the growth of *B. cinerea* likely due to the production of organic acids. The CFS of both strains was employed on artificially contaminated cut kiwifruits showing a promising capability to delay the fungal growth. Therefore, further studies should be encouraged to investigate innovative technologies to deliver antifungal metabolites from microbial origin, as well their combination with physical treatments, in order to enhance safety and shelf life without altering the overall quality of the product.

## Figures and Tables

**Table 1:** The six lactic acid bacteria (LAB) strains with best antifungal activity against *B. cinerea*.

LAB Strains	Source	Species	Reference
M04	Sourdough-C	*L. plantarum*	[18]
PAN01	Sourdough-B	*L. plantarum*	[18]
M12	Sourdough-C	*L. plantarum*	[18]
C01Rib	Sourdough-E	*L. plantarum*	[18]
UFG 121	Sourdough-A	*L. plantarum*	[28]
RIS10	Sourdough-D	*L. plantarum*	[18]

**Table 2:** pH of the CFSs obtained after 24 (CFS24) and 48 (CFS48) h of growth in MRS at 37 °C.

LAB Strains	pH	
	CFS24	CFS48
M04	3.95	3.72
PAN01	3.57	3.54
M12	3.76	3.63
C01Rib	3.65	3.56
UFG 121	3.80	3.60
RIS10	3.57	3.54

**Table 3:** pH and lactic acid (L- and D-enantiomers) production by UFG 121 and PAN01 strains monitored during 48 h of growth in MRS media.

	UFG 121						PAN01	
Time (h)	pH	Lactic Acid (g/L)	L-Lactate (g/L)	D-Lactate (g/L)	pH	Lactic Acid (g/L)	L-Lactate (g/L)	D-Lactate (g/L)
6	6.53	0.48 ± 0.07 ^dD^	0.38 ± 0.02 ^dD^	0.10 ± 0.07 ^cE^	6.54	0.49 ± 0.22 ^dD^	0.31 ± 0.25 ^cD^	0.09 ± 0.02 ^dE^
24	3.80	16.93 ± 0.55 ^cBC^	14.76 ± 0.50 ^cC^	2.17 ± 0.65 ^bBC^	3.57	14.70 ± 0.03 ^cC^	14.14 ± 0.02 ^bC^	0.56 ±0.07 ^cD^
30	3.68	20.45 ± 0.74 ^bB^	17.92 ± 0.74 ^bB^	2.53 ± 0.76 ^bB^	3.55	16.17 ± 0.19 ^bcBC^	14.89 ± 0.11 ^bC^	1.28 ± 0.46 ^bC^
48	3.60	27.18 ± 0.30 ^aA^	22.27 ± 0.32 ^aA^	4.91 ± 0.24 ^aA^	3.54	20.59 ± 0.24 ^aB^	17.94 ± 0.06 ^aB^	2.64 ± 0.72 ^aB^

Values are the average of three biological replicates. Capital letters indicate significant differences among the production of the same acid by the two strains. Lowercase letters indicate significant differences among the production of the same acid by the same strain.

## Data Availability

Please refer to suggested Data Availability Statements in section “MDPI Research Data Policies” at https://www.mdpi.com/ethics.
